# A very early steroid responder after cataract surgery: a case report

**DOI:** 10.1186/s12886-023-02991-5

**Published:** 2023-05-26

**Authors:** Atsushi Kawahara

**Affiliations:** 1 Department of Ophthalmology, Yoshida Eye Hospital, 2-31-8, Hondori, Hakodate, Hokkaido 041-0851 Japan; 2 Department of Ophthalmology, Takahashi Clinic, Kushiro, Hokkaido, Japan

**Keywords:** Steroid responder, Topical steroids, Steroid eye drops, Intraocular pressure, Cataract surgery

## Abstract

**Background:**

Increased intraocular pressure (IOP), a side effect of corticosteroid eye drops, typically develops during the first few weeks of administration, and steroid response is not generally considered a cause of increased IOP immediately after cataract surgery.

**Case presentation:**

Here, I report a rare case of increased IOP due to steroid eye drops immediately after surgery. A man in his 80s presented with vision loss. Bilateral cataracts and pseudoexfoliation syndrome were confirmed. Postoperative eye drops including steroid eye drops were started immediately after cataract surgery in the right eye. High IOP was observed at the next and subsequent morning visits, but IOP normalized when steroid eye drops were discontinued. After surgery on the left eye, steroids were not administered postoperatively, and no increase in IOP was observed.

**Conclusion:**

This case report highlights that a very early steroid response may be potential cause of elevated IOP immediately after cataract surgery.

## Background

Corticosteroid eye drops are routinely prescribed to treat and control postoperative inflammation after cataract surgery. A well-known side effect of steroid eye drops is an increase in intraocular pressure (IOP), particularly in “steroid responders” [1]. However, increases in IOP due to steroid-related side effects usually occur several weeks after the start of eye drop administration. In addition, the immediate postoperative IOP elevation is multifactorial, and it is unlikely for a steroid response to be the primary cause [2]. Herein, I report a case in which IOP was strongly suspected to have increased due to an immediate postoperative steroid response.

## Case presentation

A man in his 80s visited Takahashi Clinic with recent bilateral vision loss. There were no other associated visual symptoms. His ophthalmologic history consisted of a pterygium removed from his right eye at another clinic 10 years earlier. His family history regarding ophthalmology was unknown. His medications included amlodipine, pitavastatin calcium, and sodium ferrous citrate. He was not using eye drops.

Visual acuity tests noted a corrected distance visual acuity of 20/32 in both eyes. The IOP of the right eye measured by a non-contact tonometer was 12 mmHg and that of the left eye was 14 mmHg. Slit-lamp examination showed bilateral cataracts and pseudoexfoliation syndrome (PXF). Because the clinic was not equipped with a flare metre, it was not possible to quantify exfoliation, but slit-lamp microscopic findings were similar in the right and left eyes. Both eyes had poor mydriasis due to the PXF. In addition, the right eye showed nasal corneal opacity due to previous pterygium removal. The fundus was unremarkable. Axial lengths measured with an optical biometer were 24.43 mm for the right eye and 24.38 mm for the left eye. No preoperative visual field testing was performed.

Cataract surgery was performed on both eyes by one skilled surgeon (AK). The right eye was operated on first. After disinfection procedures were undertaken, topical anaesthesia was administered, a 2.4 mm temporal clear corneal single-plane incision was made, a sideport incision was also created, and the lens was removed by coaxial phacoemulsification. The surgery was performed using a pupil dilation ring due to poor mydriasis. A single-piece acrylic intraocular lens was inserted into the capsule bag through the unenlarged main incision. Only one cohesive ophthalmic viscosurgical device (OVD) was used during surgery. All incisions were hydrated to aid in closure after sufficient aspiration and removal of OVD from the anterior chamber, behind the iris, and behind the intraocular lens. No sutures were needed. The surgery was completed shortly after 6:00 pm and no surgical complications were observed.

After completion of the surgery, the patient was placed in a sitting position in the operating room and the nurse administered the first postoperative eye drops. The postoperative drops consisted of betamethasone sodium phosphate (steroid), moxifloxacin hydrochloride (antibiotic), and nepafenac (nonsteroidal anti-inflammatory drug). The drops were administered at 5-minute intervals. The patient was told to self-administer the drops before bedtime and the next morning before visiting the clinic. After a short rest in the recovery room, the patient was allowed to return home.

**Fig. 1:**
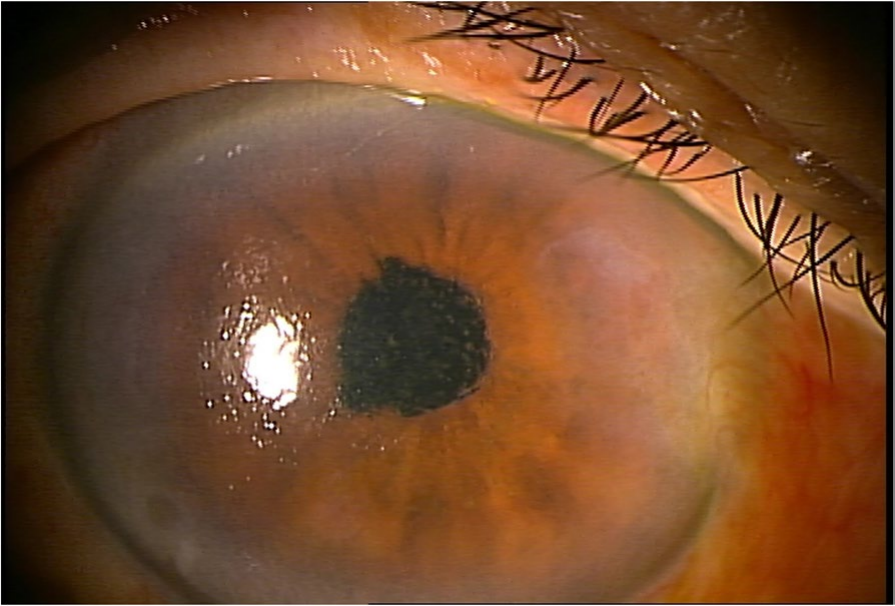
Anterior segment photograph of the right eye the morning after surgery. Corneal oedema is observed

**Fig. 2:**
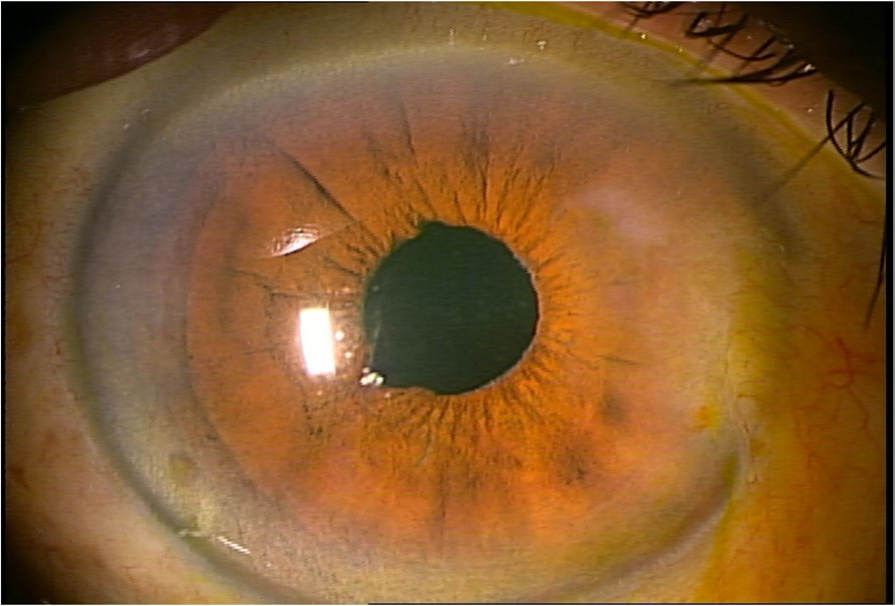
Anterior segment photograph of the right eye 1 week after surgery. Corneal oedema is resolved. The pupil remains scarred from the pupil dilation ring at the time of surgery

**Fig. 3:**
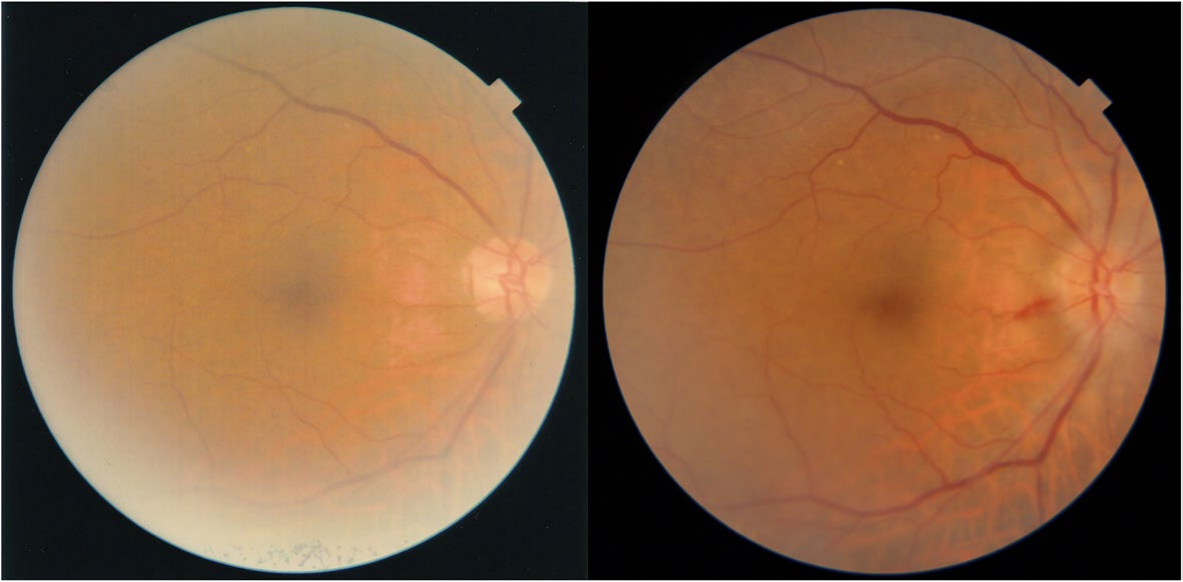
Preoperative and 1 week postoperative fundus photographs of the right eye. Optic disc haemorrhage appeared postoperatively

**Fig. 4:**
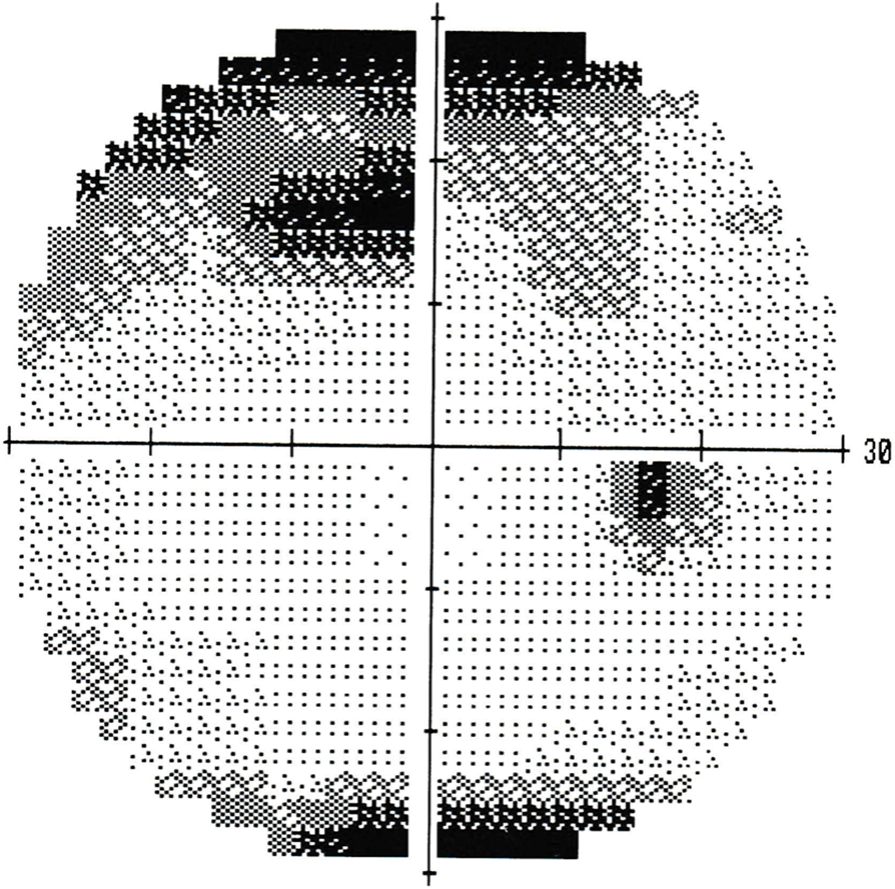
Visual field test results of the right eye 1 week after surgery. Visual field defects have appeared

The morning after surgery, the patient was examined at 9 am. The IOP in the right eye was 65 mmHg with corneal oedema (Fig. 1). Although it was difficult to identify due to corneal oedema, and convection of the anterior chamber aqueous humour was also observed. The patient reported right ocular pain. Although lowering IOP with topical and/or systemic pharmacotherapy has also been used, the posterior lip of the main incision made during surgery was pushed down with a fine needle to drain the anterior chamber aqueous humour,, which is the recommended paracentesis procedure when the postoperative IOP is 28 mmHg or higher [2]. The leaking fluid was aqueous and did not appear to be residual OVD. After the procedure, the IOP dropped to 9 mmHg. After confirming that there was no leakage of aqueous humour, from the wound by slit-lamp examination, the patient was instructed to continue eye drop treatment and to visit the doctor the next morning. When the patient was seen the next morning, the IOP was 53 mmHg and corneal oedema was again observed. The patient reported that he had begun to notice blurred vision and ocular pain after the eye drops were applied the previous afternoon. The same treatment as the previous day was performed, and the IOP was reduced to 8 mmHg. As the increase in IOP was suspected to result from a steroid response, the patient was instructed to discontinue steroid eye drops only. When he visited the clinic the next morning, his IOP was 12 mmHg and no corneal oedema was observed. As the postoperative inflammation was also reduced and appeared to be under control, the patient was instructed to return to the clinic four days later, which was the scheduled surgery date for the left eye. However, the next day, the patient came to the clinic with a main complaint of blurred vision and eye pain. The night before, the patient had mistakenly put steroid drops in his right eye, and the symptoms reappeared thereafter. The corneal oedema was mild, but the IOP was 37 mmHg. At his request, the paracentesis procedure was performed. Three days later, the patient was seen for surgery on the left eye, and the IOP of the right eye was 14 mmHg (Fig. 2). Fundus examination revealed the appearance of an optic disc haemorrhage, which was not observed preoperatively (Fig. 3). A visual field examination revealed a visual field defect (Fig. 4). The same day, left eye surgery was performed as scheduled, using the same technique as for the right eye. No surgical complications occurred. No postoperative steroid eye drops were administered, and no increase in IOP was observed in either eye after the next day. At the 1-week postoperative visit, the corrected distance visual acuity was 20/20 in both eyes, and the IOP was 12 mmHg in the right eye and 16 mmHg in the left eye. By that time, the postoperative inflammation had largely subsided. No visual field defects were observed in the left eye on visual field examination. The patient was then followed up for one month after surgery.

## Discussion and conclusions

It has been reported that early postoperative IOP elevation is observed in up to 9.8% of cataract surgeries, with risk factors including residual OVD, resident-performed surgery, glaucoma, PXF, axial length over 25 mm, tamsulosin intake, and topical steroid application in steroid responders [3]. The earliest time that increased IOP has been reported in steroid responders is the fifth postoperative day [4], and to my knowledge, there are no reports of IOP increases immediately after the administration of eye drops. Risk factors for elevated IOP due to steroid response after cataract surgery are defined as being less than 65 years of age and having an axis length of 29 mm or more; [4] however, the present case did not fall into either of these categories. Therefore, I did not consider the possibility that the patient was a steroid responder the day after surgery. It is unclear whether glaucoma developed preoperatively as no preoperative visual field testing was performed, but as the preoperative IOP was low, this case does not meet the criteria proposed by O’Brien et al. [5] who reported that a high baseline IOP is likely to cause early postoperative IOP elevation. In consideration of these facts, residual OVD and PXF were regarded as the cause. However, because convection in the anterior chamber was confirmed by slit-lamp examination, even though it was difficult to see due to corneal oedema, and the leaked anterior chamber fluid during the IOP lowering procedure did not contain anything that could be considered OVD, I later assessed PXF to be the only relevant risk factor.

According to Pohjalainen et al., [6] transient IOP peaks the day after cataract surgery are common in nonglaucomatous PXF eyes. The fact that the surgery was performed with a pupil dilation ring in this case may have contributed to an even greater release of inflammatory mediators in the eye with PXF. However, if PXF was the only cause of the early elevation of IOP in this case, the IOP should not have increased after the paracentesis procedure the day after surgery. The absence of IOP elevation after surgery in the left eye, for which the perioperative care was identical apart from the exclusion of steroid eye drops in the postoperative eye drops, proved that this patient was a steroid responder. Although IOP was not monitored, patient reports of blurred vision and eye pain appearing shortly after administration of the eye drops also support this claim. The IOP was 65 mmHg the day after surgery, 53 mmHg the following day, and 37 mmHg on the fourth postoperative day, suggesting that steroid side effects and PXF were the main causes of the increased IOP on the day after surgery and the following day, while the increase on the fourth postoperative day was due to the steroid response. The lower IOP on postoperative Day 4 may be due to the reduction in inflammatory mediators in the eye due to the paracentesis.

On the second postoperative day, I diagnosed the elevated IOP as a side effect of steroids and took early steps to lower the IOP by discontinuing steroid eye drops, but on reflection it must be noted that glaucomatous visual field defects with optic disc haemorrhage were observed one week postoperatively. However, it is not possible to determine whether the papillary haemorrhage and visual field defects were due to glaucoma or decompressive retinopathy since peripapillary retinal nerve fibre layer analysis with optical coherence tomography was not performed at the time of visual field examination. On the other hand, patients at risk of elevated IOP may be allowed to use nonsteroidal anti-inflammatory drug eye drops alone to calm postoperative inflammation as many uneventful cataract surgeries can be controlled by this treatment alone [7–10].

In this case, although I present some evidence that the IOP was not elevated due to retained OVD, this may be difficult to fully exclude, because the small pupil would make the risk of retained OVD more likely. Additionally, because of PXF, a compromised trabecular meshwork is likely. Steroids increase IOP by depositing extracellular matrix in the trabecular meshwork [4]. Therefore, it is possible that steroid, OVD, and exfoliative debris retention were imbalanced, thus leading to an increased IOP. If a flare metre had been in place at the clinic, a more detailed analysis might have been possible.

This case report indicates that elevated IOP can occur even immediately after the initiation of treatment with steroid eye drops.

## Data Availability

Data sharing is not applicable to this article as no datasets were generated or analysed during the current study.

## Abbreviations

IOP: Intraocular pressure
OVD: Ophthalmic viscosurgical device
PXF: Pseudoexfoliation syndrome
